# Correlated evolution between body size and echolocation in bats (order Chiroptera)

**DOI:** 10.1186/s12862-024-02231-4

**Published:** 2024-04-15

**Authors:** Mario G. Castro, Talita Ferreira Amado, Miguel Á. Olalla-Tárraga

**Affiliations:** 1 Departamento de Biología y Geología, Física y Química Inorgánica, Universidad Rey Juan Carlos, Calle Tulipán s/n, Móstoles, Madrid, Spain; 2 Instituto de Cambio Global, Universidad Rey Juan Carlos, Móstoles, Madrid, 28933 Spain; 3 German Center for Integrative Bioaffiliationersity Research (iDiv), Halle-Jena-Leipzig, Puschstrasse 4, 04103 Leipzig, Germany

**Keywords:** Bats, Echolocation, Body size, Allometric patterns, Correlated evolution, RevBayes

## Abstract

**Background:**

Body size and echolocation call frequencies are related in bats. However, it is unclear if this allometry applies to the entire clade. Differences have been suggested between nasal and oral emitting bats, as well as between some taxonomic families. Additionally, the scaling of other echolocation parameters, such as bandwidth and call duration, needs further testing. Moreover, it would be also interesting to test whether changes in body size have been coupled with changes in these echolocation parameters throughout bat evolution. Here, we test the scaling of peak frequency, bandwidth, and call duration with body mass using phylogenetically informed analyses for 314 bat species. We specifically tested whether all these scaling patterns differ between nasal and oral emitting bats. Then, we applied recently developed Bayesian statistical techniques based on large-scale simulations to test for the existence of correlated evolution between body mass and echolocation.

**Results:**

Our results showed that echolocation peak frequencies, bandwidth, and duration follow significant allometric patterns in both nasal and oral emitting bats. Changes in these traits seem to have been coupled across the laryngeal echolocation bats diversification. Scaling and correlated evolution analyses revealed that body mass is more related to peak frequency and call duration than to bandwidth. We exposed two non-exclusive kinds of mechanisms to explain the link between size and each of the echolocation parameters.

**Conclusions:**

The incorporation of Bayesian statistics based on large-scale simulations could be helpful for answering macroevolutionary patterns related to the coevolution of traits in bats and other taxonomic groups.

**Supplementary Information:**

The online version contains supplementary material available at 10.1186/s12862-024-02231-4.

## Background

Echolocation has long been recognized as a key trait for understanding bats’ biology [1, 2]. Along with the capacity for self-powered flight, echolocation has allowed bats to exploit the nocturnal environment. Bats use echolocation to locate, detect, and classify [3], as well as to orientate, recognize the environment, and search for food [4]. Bats can produce social calls [5], but echolocation calls can also be used as a way of communication [6]. Echolocation is highly variable among bats [7, 8], and not all species depend equally on it. The Pteropodidae family, for instance, generally lacks echolocation capacity, yet some species within the *Rousettus* genus can echolocate by generating tongue clicks [9–11]. Other pteropodids, like *Eonycteris spelaea*, can generate clicks with their wings [12]. Bat families other than Pteropodidae produce echolocation through the larynx [13]. Laryngeal echolocation is also highly diverse within bats. According to the body parts that are implied in the call emission, laryngeal echolocation can be emitted either orally or nasally [8]. Then, the variety of structure and frequencies in echolocation calls [14] has an influence on bats’ foraging ecology and diet [15, 16]. Among others, this affects the ability to forage in close or open habitats [3, 4] or the kind and size of their prey [17]. Therefore, some echolocation-call parameters such as the frequencies and duration can be seen as functional traits of bats.

Bats (order Chiroptera) are a highly diverse group of mammals with 1462 extant species, and they inhabit all continents but Antarctica [18]. They also show a wide variety of ecologies, morphologies, and behaviours [19]. Phylogenetic analyses support bats as a monophyletic group [20–22]. Bats can be divided into two major subclades: Yinpterochiroptera or Pteropodiformes (families: Pteropodidae, Hipposideridae, Rhinolophidae, Craseonycteridae, Megadermatidae, and Rhinopomatidae), and the other families would be grouped into Yangochiroptera or Vespertilioniformes [23, 24]. There is evidence supporting either a single or multiple origins of laryngeal echolocation in bats based on phylogeny, ontogeny, and the divergence in inner ear neuroanatomy [20, 25–28]. However, some studies propose a single origin based on behavioral, morphological, and neuroanatomical features [29]. Moreover, the fossil record shows early echolocating bats like Palaeochiropterygidae and Icaronycteridae [30, 31]. A recently described 50-million-year-old bat fossil appears to have used advanced laryngeal echolocation, which suggests that this capacity would have originated before the modern bat radiation [32].

Body size is known to affect the acoustic signals and vocalizations of animals [33]. Under the allometric hypothesis, size has been proposed to constrain the frequencies of animal calls, due to the negative correlation between resonation chamber size and call frequencies [34]. In laryngeal echolocating bats, predictions of the allometric hypothesis have been tested by several authors [17, 35–40]. According to this hypothesis, larger bats are expected to use lower peak frequencies (the frequency with maximum energy within an echolocation call). However, deviations from this pattern have been observed in some species [41–43], which has led to a corollary of alternative hypotheses [reviewed in 43]. This includes the evolutionary “arm race” between hearing-moths and bats [44], the use of echolocation to communicate with other bats and to reduce competition [45] and habitat-structure constraints on the performance of flight, forage, and echolocation [46]. This is a set of non-mutually exclusive hypotheses to account for the diversity of frequencies in bat calls irrespective of body size. It has been recently suggested that type of emission (nasal or oral) can lead to different peak frequency allometries, since sounds emitted by nasal structures are less variable than the ones emitted by the mouth [40]. In addition, some nasal emitting families like Phyllostomidae could not even follow the allometric hypothesis because non-insectivore diets are less dependent on echolocation [36, 40]. Body size has also been questioned by some authors as the best predictor of peak frequency, with other traits like nasal chamber size and laryngeal length suggested as alternatives [47]. To date, current knowledge on the role of type of emission on peak frequency allometries relies on a limited set of species [36, 40]. In this respect, a better insight could be gained by using a large multi-species dataset for a direct comparison of allometries in nasal and oral bats. Similarly, as we are concerned, no study has quantified the relative importance of type of emission to account for observe peak frequency allometries so far.

While peak frequency has received most of the attention in allometric studies, other echolocation parameters also have functional relevance. For example, a bat call may cover a narrow or broad range of frequencies, and this range is called the bandwidth of the call. There are also a wide variety of call durations among bat species. Peak frequency, bandwidth, and call duration have different functions and ecological consequences. First, peak frequency determines the range and detail of prey detection. Due to the quick air attenuation of high-frequency sounds, high-peak frequencies in echolocation calls can only detect insects in a short range [35]. Some bats have increased their prey-detection range by reducing their peak frequencies. Second, broad bandwidths give more detailed information of the surroundings than narrow ones [4, 35]. Last, long call durations increase the probability of detecting preys, but in cluttered spaces this can cause an overlap between emissions and reflections [3, 4]. To avoid signal overlap, some bats have evolved adaptations like Doppler shift compensation [4]. Due to flight and echolocation performance, peak frequency, bandwidth, and call duration have been linked to body size [35, 48]. In this sense, bats that use high frequencies, broad bandwidths, and short calls are expected to forage in close areas, where they must be small for a better flight maneuverability [36, 49]. Body size can also be related to bandwidth and call duration through anatomical constrains. Resonation chamber size could constrain both peak frequencies, and bandwidths [50]. Likewise, it has also been argued that call duration would be determined by lung capacity, which would be wider in larger animals, allowing them to increase the duration of their calls [39, 51]. However, the duration of bat aggressive social calls seems to be better explained by phylogenetic components than by size [50]. On the other hand, the differences in bandwidth and call duration allometries between nasal and oral emitting bats have not been addressed to our knowledge. While call duration might not be affected by type of emission, it is possible that bandwidth differs between nasal and oral emitting bats. If nasal structures constrain call frequencies making them less variable [40, 47], albeit not tested yet, the same may happen with bandwidth.

Beyond scaling patterns among extant species, understanding the evolutionary history of these echolocation functional traits and the extent to which they have co-evolved with body size can provide interesting insights into the biology of bats. Stoffberg and Jacobs [37] documented a negative correlated evolution between body mass and peak frequency within Rhinolophidae. Here, we test the extent to which changes in body size have been correlated with changes in peak frequency, bandwidth, and call duration, through the evolutionary history of laryngeal echolocating bats. Leaving aside Stoffberg and Jacobs [37], no previous study exists for a large multi-species dataset that includes different families. This test can also offer information about which echolocation parameters are more associated to body size evolution, which is especially relevant to whether body size has had a greater impact on the evolution of bandwidth or call duration.

In this study, we aim to investigate the relationship between body size and three different echolocation parameters using a large multi-species dataset of laryngeal-echolocating bats. According to the allometric hypothesis, we expect significant allometries of peak frequency, bandwidth, and call duration. However, these scaling patterns may vary between nasal and oral emitting bats [40]. If scaling patterns are a reflect of correlated evolution between echolocation and body size, we expect that body size increases would be coupled with decreases in both peak frequency and bandwidth and with increases in call duration through bat diversification. The amount of evolutionary correlation will vary between traits, indicating the different importance of size in shaping various echolocation parameters. Therefore, our objectives are (1) to assess the scaling of different echolocation parameters across the entire bat order, accounting for differences between nasal and oral emitting bats; (2) to test for correlated changes between body size and different echolocation parameters and map these changes through the diversification of the order Chiroptera; (3) to compare the importance of size shaping each of the echolocation parameters.

## Methods

We collected peak frequency, bandwidth, call duration, and body mass data from Collen [52] and removed imputed data to analyze a database of 329 bat species (Supplementary Material, Table 3). Variables were logarithmically (log10) transformed to fit a normal distribution of variables and model residuals, as required by parametric analyses. Because of echolocation calls of a bat can change as it approaches a target [3], these data were originally collected only from flights in the search phase, when bats produce less variable calls [52]. Then, given the existence of intraspecific variation in these acoustic features, several recording sequences were used to obtain a representative average for each species so it can be used for interspecific approaches [52]. We chose body mass as a measure of size since it is physiologically relevant in most mammals, and it is known to have an influence on echolocation, flight, and foraging behavior in bats, and it has been used in previous studies on allometric hypothesis and the scaling of echolocation parameters [16, 35, 36, 40]. Alternatively, some authors have used forearm length because body mass can change due to bats daily foraging [17, 19]. Body mass we collected from Collen [52] is a both sexes-averaged adult body mass (g) that can be used for comparation between animal groups in macroecological and evolutionary contexts. Despite peak frequency having received most of the attention in allometric studies, we also used bandwidth and call duration to analyze how size is related to different characteristics of echolocation. These three parameters reflect different features of echolocation that are important to describe bat calls, and comparing their scaling patterns will give us a broader perspective on how body mass is related to the evolution of echolocation. We also classified bats according to their type of emission. We classified species as nasal (Hipposideridae, Megadermatidae, Nycteridae, Phyllostomidae, Rhinolophidae, and Rhinopomatidae) or oral echolocators (Cistugidae, Craseonycteridae, Emballonuridae, Furipteridae, Miniopteridae, Molossidae, Mormoopidae, Mystacinidae, Myzopodidae, Natalidae, Noctilionidae, and Thyropteridae) according to Arbour et al. [53] to investigate whether there are scaling differences between these types of emission.

We tested whether a significant allometry exists among echolocation traits and body size, controlling for the phylogenetic relatedness between species. The three echolocation traits (peak frequency, bandwidth, and call duration) were analyzed independently by fitting a phylogenetic generalized least squares model (PGLS) with the R environment [54]. We fitted a PGLS model as a λ model of Pagel by Restricted Maximum Likelihood for each echolocation trait. This model assumes independent evolution of the traits, with a change rate proportional to the length of the branch. λ is a scaling parameter that represents the strength of the phylogenetic signal, with values ranging from 0 (trait evolution is independent of the phylogeny) to 1 (trait evolution is completely determined by the phylogeny). In each model, body mass, the additive effect of emission type, and an interaction between the two were used as predictors. The best selection of variables was determined for each model by analyzing their Akaike Information Criterion (AIC) and the model weight, applying the ‘MuMIn’ package in R [55]. Phylogenetic analyses were performed using Faurby’s phylogeny [56] with the ape and caper R packages [57, 58]. We removed all the species in the data that were not present in the phylogeny, which left 314 bat species for analysis. To estimate how each selected model fits the data, we estimated a partial R^2^ based on the likelihood of the fitted model (R^2^_lik_), which is compared to a model whose only predictor is the intercept [59]. We estimated this parameter using the rr2 package in R [60].

Differences in scaling of echolocation traits with body mass were analyzed between bat families. To do so, we selected those families that had more than twenty species in our database (Supplementary Material, Table 3). Those families were: Hipposideridae, Molossidae, Phyllostomidae, Rhinolophidae, and Vespertilionidae (32, 25, 47, 32, and 129 species, respectively; a total of 281). As before, we fitted a PGLS λ model for each echolocation parameter. Body mass, the bat’s taxonomic family, and its interactions were used as explanatory variables in each model. Then, we compared the scaling between bat families. We also estimated R^2^_lik_ for these models. The purpose of this analysis is only to add taxonomic detail to the one made between nasal and oral emitting bats.

To analyze if changes in body mass drove changes in echolocation traits across bats’ evolution, we first implemented a phylogenetic reconstruction of each variable using the ‘phytools’ package [61]. For exploratory purposes, we mapped echolocation traits (peak frequency, bandwidth, and call duration) in the phylogeny to compare them with the phylogenetic mapping of body mass. This way, we wanted to find when changes in size drove changes in echolocation through the diversification of bats and/or vice versa.

PGLS can be used to test the scaling of echolocation parameters while controlling for phylogenetic effects. However, to test correlated evolution between traits, an alternative approach is needed [62]. This will have to provide estimates of the correlation structure between traits as an indication of the extent to which body mass changes have been coupled with changes in echolocation parameters across bat evolution. Maximum likelihood or Bayesian inference can either be used to study correlated changes in traits through multivariate Brownian motion models. Bayesian inference allows to sample the posterior distribution of statistic parameters using Markov Chain Monte Carlo (MCMC). This approach allows for prior information or uncertainty about the parameters.

We tested whether there is statistical support for correlated evolution between traits using a Bayesian approach. Specifically, a multivariate Brownian Motion model (multiBM) was fitted in RevBayes [63, 64] using the parameters and instructions provided by Höhna [63, and also explained in 65]. Prior distributions were set for the average rate of change (α^2^), the relative rate of change (ζ^2^), and the correlation among characters (R). Prior distributions of these parameters for the model were estimated based on the data and the phylogenetic tree. The prior correlation is zero, and its distribution is symmetrical around this value, with the LKJ distribution (η) equal to 1. We ran 5,000,000 generations and sampled each 1000 generations. A burnin was applied, discarding the first 100,000 generations. We analyzed the correlation between traits with the ‘RevGadgets’ package on R [66]. This tool allows for the estimation of the posterior distribution of correlation densities along the 4900 sampled generations (Fig. 2). We calculated the Bayes factor, which allows us to estimate the strength and evidence with which the hypotheses are supported by the data [67]. The Bayes factor was calculated from the ratio of the posterior probabilities of the null and alternative hypotheses, with the alternative hypothesis assuming a non-zero correlation between traits. For this, we used the Savage-Dickey ratio, which compares the posterior density at zero (the null value) with the prior density at the same point.

## Results

### PGLS models for scaling tests

**Fig. 1:**
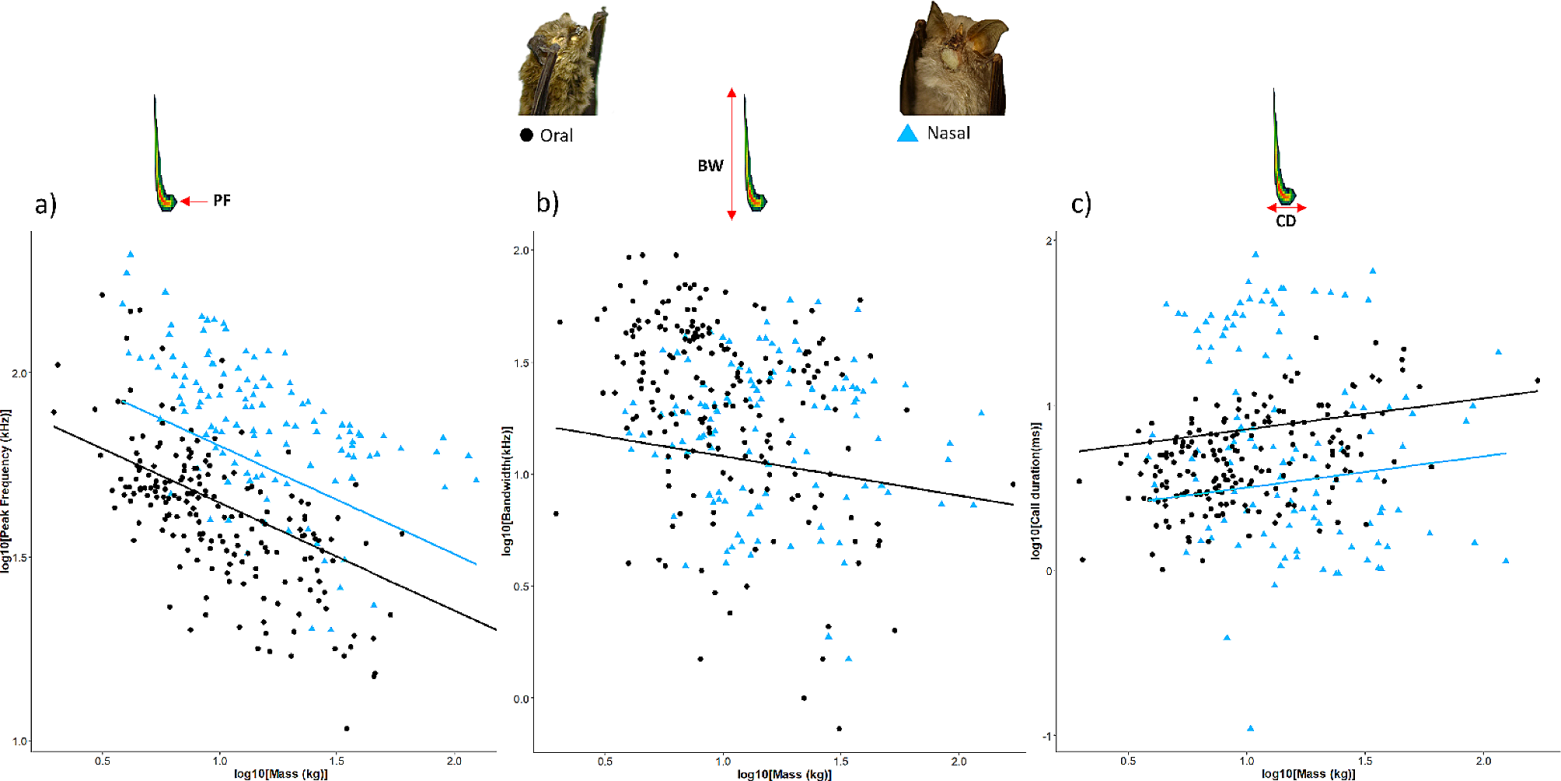
Regression fits according to the best PGLS models between body mass and peak frequency (**a**), bandwidth (**b**), and call duration (**c**). This model includes a distinction between nasal (blue triangles) and oral (black dots) emitting bats for 314 bat species. Pictures of a *Pipistrellus pipistrellus* and a *Rhinolophus hipposideros* has been included as examples of oral and nasal emitting bats respectively (MNCN-M1060 and MNCN-M357 specimens from Natural Sciences History Museum of Madrid, Spain). (PF) peak frequency, (BW) bandwidth and (CD) have been highlighted on a spectrogram of a *Pipistrellus pipistrellus* above their respective panel (bats and spectrogram images produced by M.G.C). This figure with the five major taxonomic families in the dataset distinguished can be seen in Supplementary material Fig. 1

In the analyses of scaling differences between nasal and oral bats, the best-performing PGLS model (best five models can be seen in Supplementary Material Table 1) showed a negative relationship between peak frequency and body mass (R^2^_lik_ = 0.68, slope= -0.29, C.I:-0.342, -0.244), with no significant differences in slopes between types of emission, but a higher intercept for nasal emitters (Table 1; Fig. 1a). For bandwidth, the best model only included body mass, and the association between both variables was negative (R^2^_lik_ = 0.59, slope= -0.17, C.I:-0.27,-0.08) (Table 1; Fig. 1b). We found a positive association between call duration and body mass (R^2^_lik_ = 0.70), with differences between the type of emission, and oral bats having a higher intercept (Table 1; Fig. 1c). The graphical representation of the model (Fig. 1c) showed two groups of nasal emitters. We remade this separating for the most representative families within dataset and found that the group with higher call durations corresponds with Rhinolophidae ( Supplementary Material Fig. 1).

**Table 1:** PGLS Models with the best combination of variables (i.e., lowest AIC) for the scaling of peak frequency (PF), bandwidth (BW), and call duration (CD) attending differences between nasal and oral emitters (Echo type) for 314 analyzed species. For estimating Chisq and Pr analyses of deviance type III were made, C.I. represents confidence intervals for slopes using a confidence level of 0.95%

		Value	Std error	Chisq	Pr
***PF***	*Intercept*	*2.09(nasal), 1.94(oral)*	*0.07 (nasal), 0.13(oral)*	*861.06*	*< 0.001*
	*Body mass*	*-0.29 (****C.I***:*-0.342, -0.244)*	*0.03*	*136.8*	*< 0.001*
	*Echo type*			*6.67*	*0.009*
***BW***	*Intercept*	*1.25*	*0.12*	*93.93*	*< 0.001*
	*Body mass*	*-0.18(****C.I***:*-0.27,-0.08)*	*0.06*	*13.19*	*< 0.001*
			*0.36*		
***CD***	*Intercept*	*0.31 (nasal), 0.55(oral)*	*0.14*	*5.23*	*0.02*
	*Body mass*	*0.18 (****C.I***: *0.20, 0.27)*	*0.05*	*16.59*	*< 0.001*
	*Echo type*		*0.11*	*9.44*	*0.002*
			*0.33*		

When analyzing scaling differences between families, we found that the best performing PGLS model for peak frequency only included body mass, with a slope of -0.269 (R^2^_lik_ = 0.41, C.I. = -0.33, -0.21) (Supplementary Material Table 2). For bandwidth, there were significant differences in the y-intercepts between families, with Vespertilionidae having a higher intercept than the others. However, all families scaled with a slope of -0.193 (R^2^_lik_ = 0.55, C.I. = -0.29, -0.10) (Supplementary Material Table 2). Finally, for call duration, the scaling was also the same for all families, with a slope of 0.184 (R^2^_lik_ = 0.77, C.I: 0.09, 0.28). There were differences in the y-intercepts for Rhinolophidae and Phyllostomidae compared to the rest of the families, with Rhinolophidae having longer call durations and Phyllostomidae having shorter call durations for their size (Supplementary Material Fig. 1c).

### Correlated evolution

**Fig. 2:**
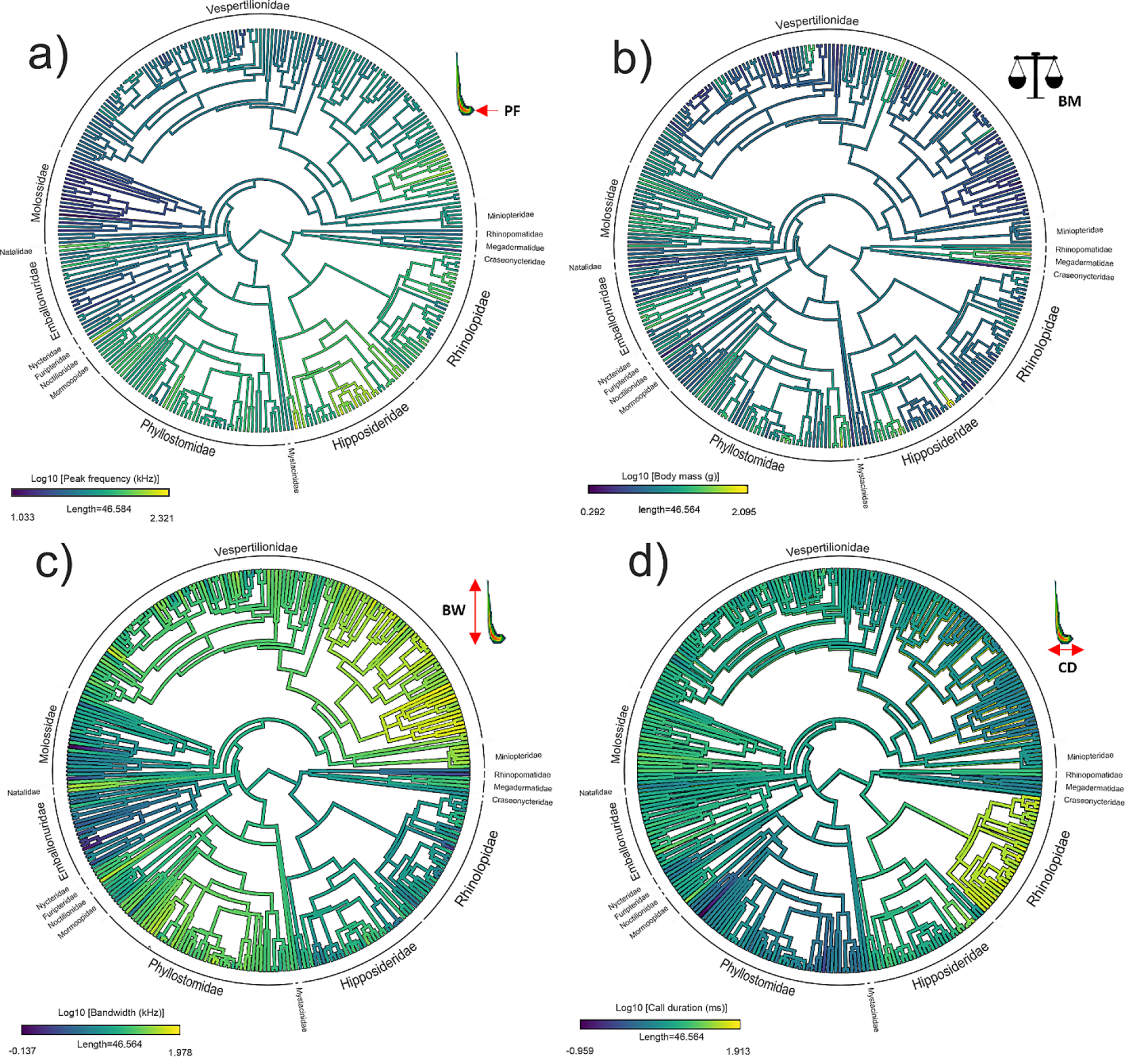
Phylogenetic mapping for 314 and 15 bat species and families, respectively, of **a**) echolocation peak frequency (PF), **b**) body mass (BM), **c**) echolocation bandwidth (BW), and **d**) call duration (CD). All variables are represented as log-transformed due to the non-normal distribution of data. Spectrograms of a *Pipistrellus pipistrellus* call highlighting each trait were included in their respective panel to help visualization

**Fig. 3:**
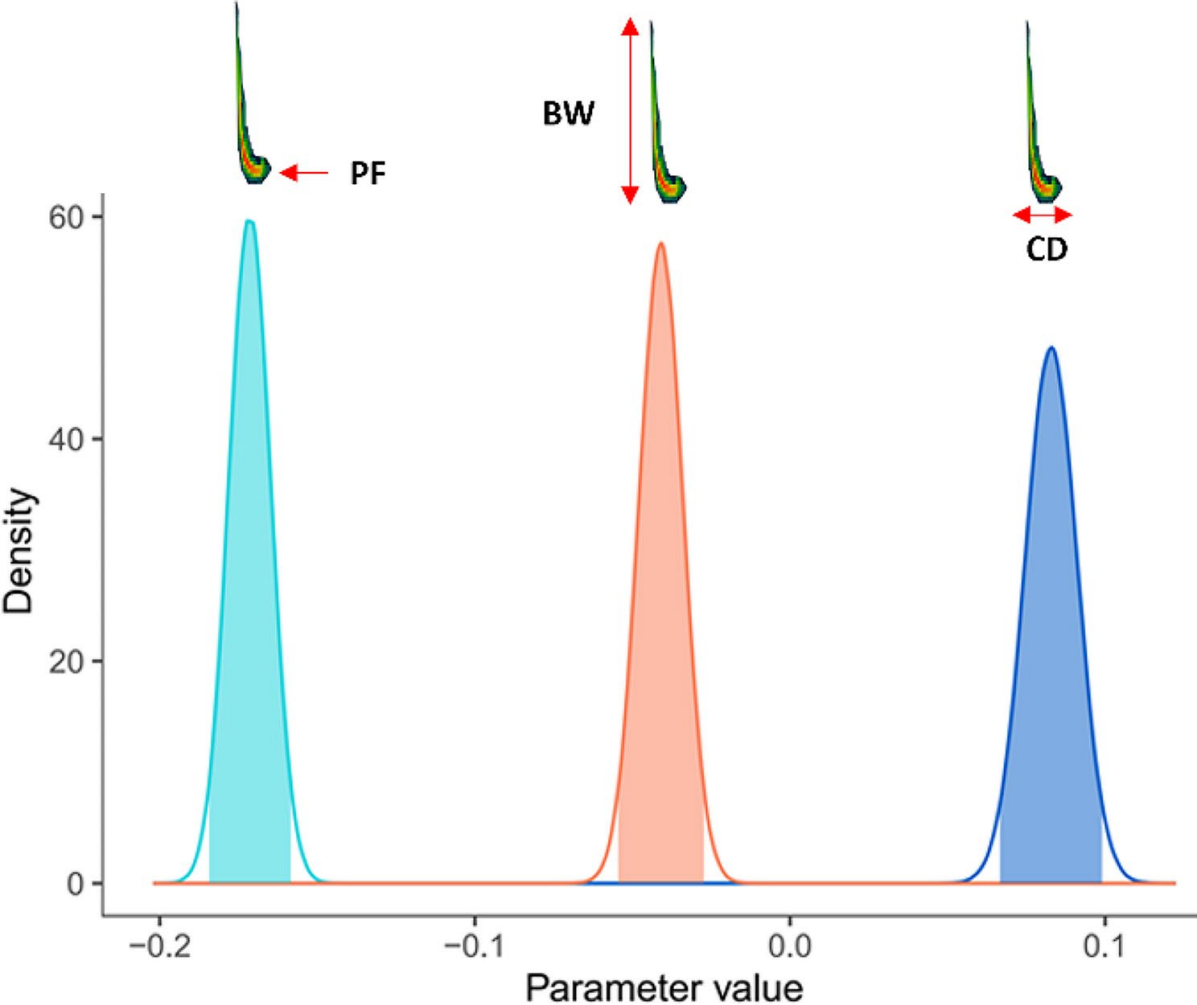
Evolutionary correlations between body mass and the echolocation parameters; from left to right: peak frequency, bandwidth, and call duration. Density represents the estimate of the posterior distribution of correlation densities along the 4900 sampled generations of 5,000,000 generations that were run in RevBayes.

Through trait mapping on the phylogeny of bats (Fig. 2), we noted that increases in peak frequency (Fig. 2a) were associated with decreases in body mass (Fig. 2b) in Vespertilionidae, Miniopteridae, Natallidae, Emballonuridae, Hipposideridae, and Rhinolophidae. Conversely, decreases in peak frequency were associated with body size increases in Molossidae. This negative relationship between body mass and peak frequency given by visual exploration was confirmed by Bayesian analyses, which detected a negative correlated evolution between peak frequency and body mass (Fig. 3). The density of correlations estimated across all MCMC simulations had a range of -0.18 to -0.16 and a maximum density value of -0.17 for the correlation between peak frequency and body mass. Bayes factor had a value of 39.38.

For bandwidth (Fig. 2c), trait mapping suggested a negative correlation with body mass (Fig. 2b), particularly in Vespertilionidae, Natalidae, Phyllostomidae, and Emballonuridae. Bayesian analysis (Fig. 3) also confirmed the existence of a negative correlated evolution between bandwidth and body mass, with a range of -0.05 to -0.03 and a maximum density value of -0.04, supported by a Bayes factor of 37.80.

Finally, trait mapping indicated a positive association between call duration (Fig. 2d) and body mass (Fig. 2b) in some families, such as Miniopteridae, Vespertilionidae, Molossidae, and especially Rhinolophidae. In agreement with this, Bayesian analysis documented a positive correlated evolution between call duration and body mass for the whole order, with a range of 0.07 to 0.1 and a maximum density value of 0.085, supported by a Bayes factor of 30.93.1.

In sum, both trait co-evolution analyses based on trait mapping in the phylogeny (Fig. 2) and Bayesian statistics (Fig. 3) provided consistent evidence for a negative correlated evolution between peak frequency and body mass, as well as for bandwidth and body mass, and a positive correlation for call duration and body mass. Bandwidth had correlation values closer to zero than the other traits (Fig. 3). The strong support for these correlations in the data suggests that they are likely to be robust across different taxa of bats. Bayes factor above 30 indicated strong support for these correlations in our data [68].

## Discussion

We found support for correlated evolution of peak frequency, bandwidth, and call duration with body mass. As expected, increases in size were coupled with decreases in both peak frequency and bandwidth and with increases in call duration (and vice versa) through bat diversification. Bandwidth posterior correlation distributions were the closest ones to zero (Fig. 3), which suggests weaker correlated evolution for this echolocation parameter. These results were congruent with the testing of allometries, in which significant scaling patterns were detected for all three echolocation parameters. Our analyses identified higher prediction power (R^2^_lik_) of body size on call duration, followed by peak frequency and bandwidth. These results are congruent with other studies [37], who found that changes in peak frequency were correlated with body size in Rhinolophidae evolution over changes in habitat type. Our results expand this finding for a large interfamily database, suggesting that this correlation might have been global for laryngeal echolocating bats.

The mechanisms that would explain the allometric patterns, and correlated evolution of the different echolocation parameters with body size, could rely on anatomical constrains driven by size, that would not be exclusive for bats. For example, as expected by the allometric hypothesis, increases in body size influences resonation chambers size, making larger animals constrained to produce lower-frequency sounds [34, 69]. This includes bats echolocation peak frequency. We found evidence of correlated evolution between body mass and peak frequency and a significant global allometry close to -0.3 between these traits on a logarithmic scale. This finding is in consonance with the pattern expected under the allometric hypothesis [17, 36–40]. In addition, albeit all call parameters exhibited correlated evolution, we found that during bat diversification body mass changes were more coupled with changes in call duration than with those of bandwidth. Moreover, when analyzing the allometric patterns of these echolocation parameters, PGLS R^2^_lik_ for bandwidth were also lower than for call duration. While increases in body size may increase the air capacity of the lungs allowing larger animals to produce longer calls [39, 51], morphological constrains of size to bandwidth are less clear. Perhaps the constrains of the later would be related with the ones of peak frequency (i.e., bandwidth would be restricted by resonation chamber size, similarly to peak frequency) [50]. Overall, our results suggest that bandwidth (compared to peak frequency and call duration) could be more explained by factors other than body size (e.g., other morphological constraints, recording methods, diet, habitat structure, and behavior; [3, 4, 45, 46]).

There are other mechanisms to explain the scaling and coevolution patterns between peak frequency, bandwidth, call duration and body size. These mechanisms would rely on flight and echolocation performance constrains and are exclusive of bats. In this sense, it has been even suggested that echolocation imposed a selective pressure on bats size, and that is why they would be small [35, 48]. Because of the quick air attenuation of high-frequency sounds, and the weak signal given by small insects, the high-peak frequencies of echolocation calls can only detect these insects in a short range. Small bats with rounded wings will be better for this and will perform better foraging within a cluttered environment [4], where they do not only have to forage but to avoid obstacles in darkness. In this environment a detailed information of the surroundings given by broadband calls (high bandwidth) is needed, yet an overlap between emitted and received signals must be avoided by making calls shorter [35]. A relatively big bat which fly faster and is less manurable will perform better foraging in open areas [4]. In this scenario, to avoid the attenuation of echolocation and to increase the range of prey and/or obstacle detection, the frequency of the calls must be reduced [35]. In an open area, detailed information of the surroundings is not needed but prey must be detected within a wider space, otherwise, an overlap of calls is less likely to occur. Therefore, calls must be focused on the frequencies which allow insect detection (using short bandwidths) and can be longer than in a cluttered environment (increasing call duration). Some interesting exceptions to this can be observed in some bats. Firstly, there are bats with doppler shift compensation capacity like Rhinolophidae (also found in Hipposideridae and some Mormoopidae), which do not suffer from the overlap and have long calls while foraging in cluttered habitats. As can be seen in Supplementary Material Fig. 1, Rhinolophidae have longer calls than expected from their size. Increasing call intensity could be an alternative strategy to deal with air attenuation, which would be especially beneficial for open foragers. Although relatively larger animals like open foragers should afford the cost of increasing call intensity, the body size-call intensity relationship has been found non linear in terrestrial environments [70]. Currie et al. [71] documented the high cost of high intensity calls in bats. This may have resulted in the development of alternatives to compensate for atmospheric attenuation, such as increasing call duration and reducing frequencies. This statement would be congruent with our results. However, to test this hypothesis, future research should compare the metabolic costs of increasing intensity versus increasing call duration. It is also needed to look at the mode of evolution of call intensity and its correlation with the other acoustic parameters and body size. Frugivorous and nectarivore bats may have been able to overcome the echolocation constrain of size by utilizing other senses like vision and olfaction in foraging [48]. Pteropodidae (family with the biggest species of the Chiroptera order) could have even lost laryngeal echolocation, potentially allowing them to increase in size [48]. Other alternatives to body size have been proposed to modify and explain the diversity of bat echolocation calls. First, the co-evolution with moths that can hear bat calls seems to alter the frequencies with whom some bats forage [17, 44, 72]. Second, the role of echolocation in communication has been proposed to be useful in niche partitioning and reducing competition inter and intra-specifically [45]. Then, how this may affect echolocation call-structures is a still an important research area due to the functional-ecological meaning of these.

The allometries of the three echolocation parameters were significant. Additionally, for peak frequency and call duration, the best model not only included body mass but also an additive effect of emission type (nasal or oral). This model was preferred over a model with the interaction of body mass and emission type, or another one with body mass only (Table 1; Supplementary Material Table 1). The best model for bandwidth only included body mass, meaning that emission type was not as relevant to explain this parameter variation. Therefore, our results support that the allometric hypothesis predicted pattern is globally applicable for both, nasal and oral emitting bats. We initially expected to find differences in the allometric slopes between types of emission. The reasoning for this was that peak frequencies of nasal emitting bats could be more determined by nasal structures such as nose morphology than by resonation chamber sizes [47]. Then, this could lead to significant differences in the allometries of nasal and oral emitting bats. According to our results, peak frequency of nasal emitting bats could be dependent on the resonation chamber size too. However, nasal structures like nose leaves or nasal resonators produce higher frequencies than oral emission, according to the higher intercepts found in our study (see Fig. 1). These higher frequencies for nasal emitting bats could be the result of an adaptation to enhance the directionality of the calls [73]. The ultrasound produced in the larynx of a nasal emitting bat without nose-leaves would lose directionality quickly due to air absorption of the sound [74]. To overcome this issue, nasal emitting bats could have developed nose leaves and higher-frequency calls [73]. Likewise, we expected a variation between nasal and oral emission in the scaling of bandwidth too. If nasal structures constrain the variability of call frequencies, they could also constrain bandwidth itself [40]. We found that there were not significant scaling differences between types of emission for call duration. All nasal emitters but Rhinolophidae had shorter calls compared to oral emitters. Rhinolophidae, had significantly longer calls due to their calls with Doppler compensation (a characteristic also found in Hipposideridae and some Mormoopidae), which allowed them to produce longer calls without interference [75]. All these results expand previous works on the allometric differences between nasal and oral emitting bats [40], while encouraging further investigation into the scaling of nose leaves and nasal cavities and the effect of this on call emission. The morphology of nasal structures seems to have a functional ecological meaning, as suggested for Phyllostomidae and Rhinolophidae [76–78]. Phyllostomidae represents an interesting group to explore the evolution of sensory systems, morphology, and dietary niches. These neotropical bats show a wide diversity of diets from pollen, nectar, fruits and foliage to insects, vertebrates (including other bats) and blood. In this regard, it has been documented how changes in sensorial systems given by opportunity and modularity of their underlying morphological structures allowed the access to novel diet and ecological opportunities for Phyllostomidae, and this led to their interesting ecomorphological diversity and radiation [79, 80]. Further research on morphological aspects related to sensorial ecology like nose-leaves can still contribute to a more in-depth understanding of bat diversity and evolution.

Our study is the first one to address correlated evolution in bats using RevBayes, a recently developed Bayesian approach based on large scale simulations. This is a valuable tool complementary to more traditional approaches like PGLs to test patterns like those proposed by the allometric hypothesis. One of the differences between RevBayes and PGLs is that the former can test whether, and how much, changes in a trait have been coupled with changes in other traits throughout the diversification of a given taxon. However, this Bayesian tool has limitations, such as the inability to combine quantitative and categorical variables in the analysis. For example, this combination would be useful for testing the evolutionary correlation between size and echolocation parameters while assessing the differences between nasal and oral emitting bats, foraging habitats, diets, or families. Even so, RevBayes is still interesting for adding more evolutionary context in the exploration of biological questions regarding the relationships between functional traits. Specifically for our purposes, this Bayesian approach has provided a comprehensive perspective of echolocation and body size correlated evolution across a large number of laryngeal echolocating bat species.

## Conclusions

In this work, our aim was to address the relationship between echolocation and body size in bats. Firstly, by testing scaling patterns of peak frequency, bandwidth, and call duration, while directly accounting for differences between nasal and oral emitting bats. Secondly, by analyzing whether there has been a correlated evolution between size and these echolocation parameters. Lastly, by comparing the influence of size on each of these parameters. According to our results, larger bats tend to have lower frequencies, narrower bands, and longer call durations, as expected. However, significant differences between nasal and oral emitting bats on scaling intercepts for peak frequency and call duration were included in the best selected PGLS models. We did not find significant differences in slopes scaling between nasal and oral emitting bats for any of the echolocation parameters. There was not any sort of differences in scaling patterns of bandwidth between types of emission. All of this expands previous studies findings on the echolocation allometric differences between types of emission [40]. In this study we detected that increases in body mass were coupled with decreases in peak frequency and bandwidth and with increases in call duration (and vice versa) across laryngeal-echolocating bat diversification (as tested by using RevBayes analyses and explored by phylogenetic mapping). Both approaches (PGLS and RevBayes) found that the relationship of body mass with peak frequency and call duration was greater than with bandwidth. We discussed two alternatives, but not exclusive, kinds of mechanisms to explain the constraints between size and echolocation. One is based on morphology and anatomy, and the other on flight, echolocation performance and habitat structure. All of these may explain not only the allometric patterns seen in extant bats but also the correlated evolution between them. The flight-echolocation-morphology complex has an enormous influence on bat foraging strategies and ecology. Body size, peak frequency, bandwidth, and call duration can be seen as functional traits for bats. The diversity of foraging strategies and their evolutionary link with sensory ecology still deserve further research. Our study adds evidence that incorporating large-scale simulation analyses, phylogenetic reconstruction, and Bayesian statistics, can benefit the exploration of macroevolutionary patterns.

### Electronic supplementary material

Below is the link to the electronic supplementary material.

Supplementary Material 1

## Data Availability

Data used in this study was adapted from Collen (2012), available as open access at UCL Discovery. All the modifications of this are detailed in methods sections. Derived datasets will be uploaded to Zenodo and the DOI of the repository will be provided upon manuscript acceptance. Main database is also provided in Table 3 of Supplementary Material.
