# Supplementary material for: Correlated evolution between body size and echolocation in bats (order Chiroptera)
Source: BMC Ecol Evol. 2024 Apr 15;24:44. doi: 10.1186/s12862-024-02231-4 (PMC11017568; doi:10.1186/s12862-024-02231-4)
Supplement: Supplementary file 1 — Supplementary Material 1 [file 12862_2024_2231_MOESM1_ESM.docx]

Supplementary Material

**Table 1:** Model selection according to their AICc and weight for the scaling of each echolocation parameter. Peak frequency (PF) bandwidth (Band) and call duration (Call Dur). Ech. Type is the type of echolocation (i.e. nasal or oral) BM is body mass and Ech. Type:BM is the interaction between echolocation type body mass. Analyses done for 314 bat species. Significancy *P* codes: 0 ‘***’ 0.001 ‘**’ 0.01 ‘*’ 0.05 ‘.’ 0.1 ‘ ’ 1

| Model | Int | Ech.T | BM | Ech.T: BM | df | logLik | AICc | weight |
| --- | --- | --- | --- | --- | --- | --- | --- | --- |
| PF |  |  |  |  |  |  |  |  |
| 4 | 2.094 | +^**^ | -0.293^***^ | _ | 5 | 202.62 | -395.00 | 0.54 |
| 3 | 1.999 | _ | -0.284^***^ | _ | 4 | 201.36 | -394.60 | 0.43 |
| 8 | 2.107 | +^*^ | -0.303^***^ | + | 6 | 200.63 | -389.00 | 0.03 |
| 1 | 1.682 | _ | _ | _ | 3 | 151.36 | -296.60 | 0.00 |
| 2 | 1.718 | + | _ | _ | 4 | 150.00 | -292.00 | 0.00 |
|  |  |  |  |  |  |  |  |  |
|  |  |  |  |  |  |  |  |  |
| Band |  |  |  |  |  |  |  |  |
| 3 | 1.256 | _ | -0.176^***^ | _ | 4 | -1.87 | 11.90 | 0.72 |
| 4 | 1.364 | + | -0.185^***^ | _ | 5 | -1.99 | 14.20 | 0.23 |
| 1 | 1.059 | _ | _ | _ | 3 | -6.10 | 18.30 | 0.03 |
| 8 | 1.393 | + | -0.208^**^ | + | 6 | -3.29 | 18.90 | 0.02 |
| 2 | 1.129 | + | _ | _ | 4 | -6.75 | 21.60 | 0.01 |
|  |  |  |  |  |  |  |  |  |
|  |  |  |  |  |  |  |  |  |
| Call Dur |  |  |  |  |  |  |  |  |
| 4 | 0.313 | +^**^ | 0.189^***^ | _ | 5 | 12.23 | -14.30 | 0.75 |
| 8 | 0.403 | + | 0.115 | + | 6 | 11.79 | -11.30 | 0.17 |
| 3 | 0.524 | _ | 0.172^***^ | _ | 4 | 8.89 | -9.70 | 0.07 |
| 2 | 0.551 | + | _ | _ | 4 | 6.35 | -4.60 | 0.01 |
| 1 | 0.715 | _ | _ | _ | 3 | 4.44 | -2.80 | 0.00 |

**Table 2:** Model selection according to their AICc and weight for the scaling of each echolocation parameter. Peak frequency (PF) bandwidth (Band) and call duration (Call Dur). Fam is bat taxonomic family analyses were run for families with at least 20 species within the database ( Hipposideridae, Molossidae, Phyllostomidae, Rhinolophidae and Vespertilionidae). BM is body mass and Fam:BM is the interaction between taxonomic family and body mass. Analyses done for 265 bat species. Significancy *P* codes: 0 ‘***’ 0.001 ‘**’ 0.01 ‘*’ 0.05 ‘.’ 0.1 ‘ ’ 1

| Model | Int | Fam | BM | Fam: BM | df | logLik | AICc | weight |
| --- | --- | --- | --- | --- | --- | --- | --- | --- |
| PF |  |  |  |  |  |  |  |  |
| 3 | 2.018 | _ | -0.269^***^ | _ | 3 | 88.14 | -170.20 | 0.96 |
| 4 | 2.263 | + | -0.268^***^ | _ | 7 | 88.71 | -163.00 | 0.03 |
| 8 | 2.184 | + | -0.194^**^ | +^***^ | 11 | 92.69 | -162.30 | 0.02 |
| 1 | 1.703 | _ | _ | _ | 2 | 54.42 | -104.80 | 0.00 |
| 2 | 1.976 | + | _ | _ | 6 | 55.67 | -99.00 | 0.00 |
|  |  |  |  |  |  |  |  |  |
|  |  |  |  |  |  |  |  |  |
| Band |  |  |  |  |  |  |  |  |
| 4 | 1.288 | +^***^ | -0.193^***^ | _ | 8 | 13.88 | -11.20 | 0.51 |
| 3 | 1.394 | _ | -0.205^***^ | _ | 4 | 8.96 | -9.80 | 0.25 |
| 8 | 1.177 | + | -0.091 | +^**^ | 12 | 17.38 | -9.50 | 0.22 |
| 2 | 1.079 | + | _ | _ | 7 | 8.88 | -3.30 | 0.01 |
| 1 | 1.156 | _ | _ | _ | 3 | 3.53 | -1.00 | 0.00 |
|  |  |  |  |  |  |  |  |  |
|  |  |  |  |  |  |  |  |  |
| Call Dur |  |  |  |  |  |  |  |  |
| 4 | 0.671 | +^***^ | 0.184^***^ | _ | 8 | 31.13 | -45.70 | 0.98 |
| 2 | 0.870 | +^***^ | _ | _ | 7 | 25.91 | -37.40 | 0.02 |
| 8 | 0.645 | +^*^ | 0.207^*^ | + | 12 | 28.84 | -32.40 | 0.00 |
| 3 | 0.585 | _ | 0.183^***^ | _ | 4 | 17.41 | -26.70 | 0.00 |
| 1 | 0.797 | _ | _ | _ | 3 | 12.97 | -19.90 | 0.00 |


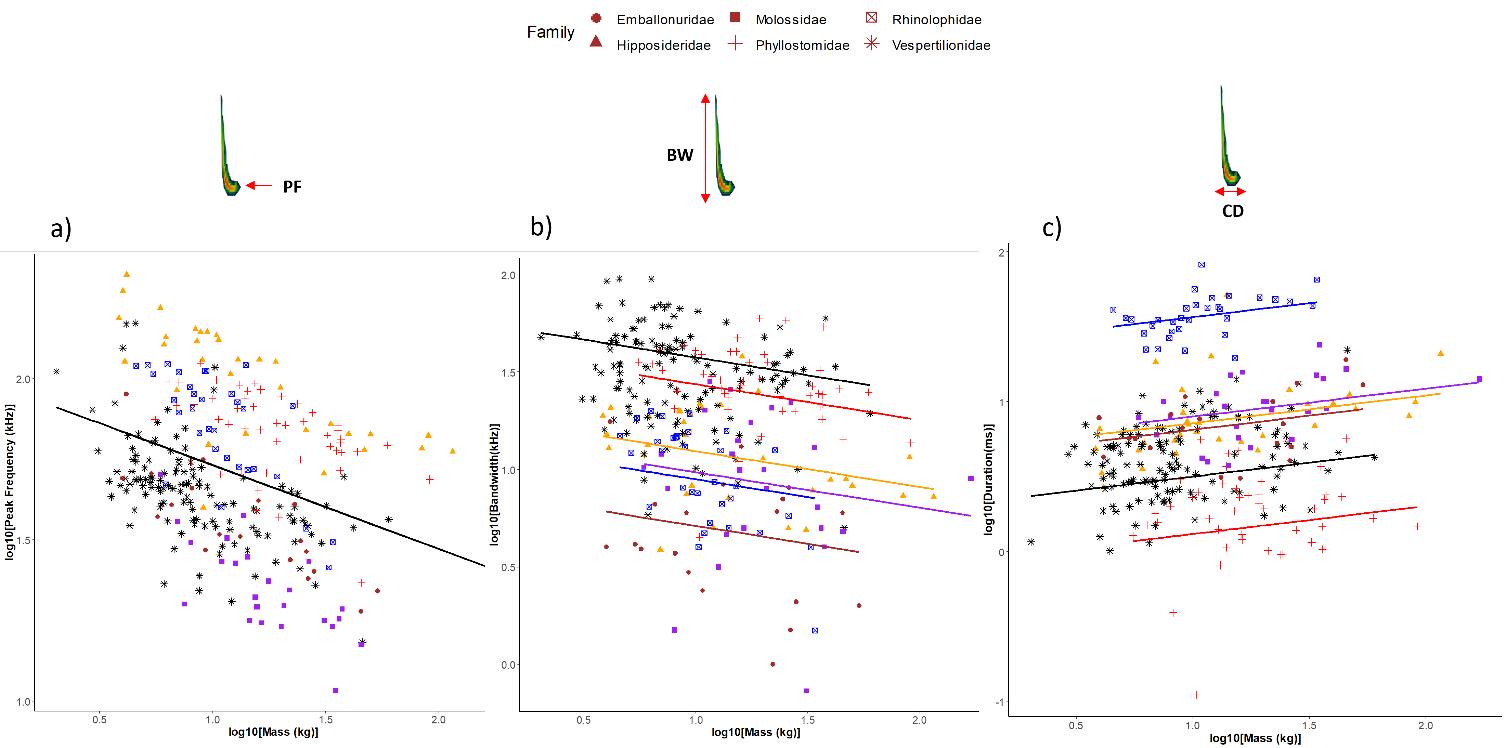


**Figure 1:** Regression fits according to the best PGLS models between body mass and peak frequency (a), bandwidth (b), and call duration (c). Comparing slopes (left) between six taxonomic families of bats (brown dots: Emballonuridae; yellow triangles: Hipposideridae; purple squares: Molossidae; red crosses: Phyllostomidae; crossed squares: Rhinolophidae; and black stars: Vespertilionidae) for 281 species. See details of this model in Supplementary material

**Table 3:** Main database used in the study. (Band) Bandwidth (KHz); (BM) body mass (g); (Call Dur); call duration (ms); (PF) peak frequency and (Ech. T) echolocation type (i.e nasal and oral). Taken from measured data in Collen (2012), bat species were classified as oral or nasal according to Arbour et al. (2021). Taxonomy validated at Integrated Taxonomic Information System (itis.gov)

| **Suborder** | **Family** | ***Species*** | **Band** | **BM** | **Call Dur** | **PF** | **Ech.T** |
| --- | --- | --- | --- | --- | --- | --- | --- |
| **YingPterochiroptera** | Craseonycteridae | *Craseonycteris thonglongyai* | 6.64 | 1.96 | 3.48 | 78.17 | oral |
| **Yangochiroptera** | Emballonuridae | *Balantiopteryx infusca* | 6 | 9.05 | 6.7 | 56 | oral |
| **Yangochiroptera** | Emballonuridae | *Balantiopteryx io* | 4 | 3.97 | 7.8 | 49 | oral |
| **Yangochiroptera** | Emballonuridae | *Balantiopteryx plicata* | 6.66 | 6.57 | 4.93 | 40.57 | oral |
| **Yangochiroptera** | Emballonuridae | *Centronycteris maximiliani* | 6.1 | 23 | 5.29 | 40.8 | oral |
| **Yangochiroptera** | Emballonuridae | *Coleura afra* | 2.4 | 10.68 | 7.7 | 32.9 | oral |
| **Yangochiroptera** | Emballonuridae | *Cormura brevirostris* | 2.96 | 9.26 | 10.8 | 29.38 | oral |
| **Yangochiroptera** | Emballonuridae | *Emballonura dianae* | 8.38 | 13.21 | 3.13 | 32.36 | oral |
| **Yangochiroptera** | Emballonuridae | *Emballonura furax* | 13.09 | 15.94 | 3.08 | 41.9 | oral |
| **Yangochiroptera** | Emballonuridae | *Emballonura monticola* | 4.15 | 5.35 | 5.68 | 50.76 | oral |
| **Yangochiroptera** | Emballonuridae | *Peropteryx macrotis* | 3.9 | 5.68 | 5.63 | 37.35 | oral |
| **Yangochiroptera** | Emballonuridae | *Rhynchonycteris naso* | 17.6 | 4.14 | 4.25 | 89.69 | oral |
| **Yangochiroptera** | Emballonuridae | *Saccolaimus flaviventris* | 6 | 45.25 | 19 | 19 | oral |
| **Yangochiroptera** | Emballonuridae | *Saccopteryx bilineata* | 3.71 | 8.08 | 8.27 | 45.76 | oral |
| **Yangochiroptera** | Emballonuridae | *Taphozous hilli* | 1 | 21.99 | 10 | 27.5 | oral |
| **Yangochiroptera** | Emballonuridae | *Taphozous kapalgensis* | 1.5 | 26.45 | 5 | 24 | oral |
| **Yangochiroptera** | Emballonuridae | *Taphozous mauritianus* | 2.09 | 27.97 | 13.27 | 25.29 | oral |
| **Yangochiroptera** | Emballonuridae | *Taphozous melanopogon* | 8.05 | 25.99 | 4.06 | 29.11 | oral |
| **Yangochiroptera** | Emballonuridae | *Taphozous perforatus* | 7.09 | 24.43 | 7.1 | 31.38 | oral |
| **Yangochiroptera** | Emballonuridae | *Taphozous troughtoni* | 2 | 53.52 | 13 | 22 | oral |
| **Yangochiroptera** | Furipteridae | *Furipterus horrens* | 54.39 | 3.15 | 2.74 | 162.68 | oral |
| **YingPterochiroptera** | Hipposideridae | *Asellia tridens* | 21.46 | 12.94 | 5.57 | 114.22 | nasal |
| **YingPterochiroptera** | Hipposideridae | *Aselliscus stoliczkanus* | 13.34 | 6.09 | 3.55 | 127.19 | nasal |
| **YingPterochiroptera** | Hipposideridae | *Aselliscus tricuspidatus* | 12.81 | 4.08 | 3.31 | 112.82 | nasal |
| **YingPterochiroptera** | Hipposideridae | *Cloeotis percivali* | 20.74 | 4.16 | 2.67 | 209.31 | nasal |
| **YingPterochiroptera** | Hipposideridae | *Hipposideros armiger* | 8.24 | 49.99 | 8.87 | 67.07 | nasal |
| **YingPterochiroptera** | Hipposideridae | *Hipposideros ater* | 11.88 | 5.86 | 4.72 | 165.4 | nasal |
| **YingPterochiroptera** | Hipposideridae | *Hipposideros bicolor* | 20.06 | 8.39 | 5.51 | 142.18 | nasal |
| **YingPterochiroptera** | Hipposideridae | *Hipposideros caffer* | 18.94 | 9.46 | 7.38 | 139.32 | nasal |
| **YingPterochiroptera** | Hipposideridae | *Hipposideros calcaratus* | 25.26 | 18.99 | 4.72 | 112.71 | nasal |
| **YingPterochiroptera** | Hipposideridae | *Hipposideros cervinus* | 19.86 | 8.51 | 4.48 | 130.37 | nasal |
| **YingPterochiroptera** | Hipposideridae | *Hipposideros cineraceus* | 18.83 | 3.84 | 4.85 | 153.59 | nasal |
| **YingPterochiroptera** | Hipposideridae | *Hipposideros commersoni* | 11.57 | 89.99 | 9.94 | 66.43 | nasal |
| **YingPterochiroptera** | Hipposideridae | *Hipposideros diadema* | 8.89 | 46.9 | 11.12 | 59.98 | nasal |
| **YingPterochiroptera** | Hipposideridae | *Hipposideros fulvus* | 17.45 | 8.83 | 5.98 | 138.99 | nasal |
| **YingPterochiroptera** | Hipposideridae | *Macronycteris gigas* | 7.24 | 115.4 | 20.9 | 59.28 | nasal |
| **YingPterochiroptera** | Hipposideridae | *Hipposideros halophyllus* | 15.16 | 4 | 6.6 | 186.92 | nasal |
| **YingPterochiroptera** | Hipposideridae | *Hipposideros inornatus* | 5 | 25.9 | 12 | 69.2 | nasal |
| **YingPterochiroptera** | Hipposideridae | *Hipposideros larvatus* | 11.4 | 19.95 | 5.87 | 93.57 | nasal |
| **YingPterochiroptera** | Hipposideridae | *Hipposideros lekaguli* | 4.91 | 31.08 | 9.6 | 50.71 | nasal |
| **YingPterochiroptera** | Hipposideridae | *Hipposideros lylei* | 8.85 | 40 | 9.65 | 67.27 | nasal |
| **YingPterochiroptera** | Hipposideridae | *Hipposideros maggietaylorae* | 38.09 | 16.1 | 5.37 | 114.26 | nasal |
| **YingPterochiroptera** | Hipposideridae | *Hipposideros pomona* | 18.57 | 6.2 | 5.27 | 134.3 | nasal |
| **YingPterochiroptera** | Hipposideridae | *Hipposideros pratti* | 7.35 | 84.38 | 8.01 | 60.77 | nasal |
| **YingPterochiroptera** | Hipposideridae | *Hipposideros ridleyi* | 8.26 | 9.59 | 7.06 | 62.36 | nasal |
| **YingPterochiroptera** | Hipposideridae | *Hipposideros ruber* | 20.86 | 10.61 | 7.22 | 131.51 | nasal |
| **YingPterochiroptera** | Hipposideridae | *Hipposideros semoni* | 7.13 | 14 | 51.43 | 67.46 | nasal |
| **YingPterochiroptera** | Hipposideridae | *Hipposideros speoris* | 21.77 | 10.39 | 6.29 | 135.74 | nasal |
| **YingPterochiroptera** | Hipposideridae | *Doryrhina stenotis* | 5 | 12 | 20 | 103 | nasal |
| **YingPterochiroptera** | Hipposideridae | *Hipposideros turpis* | 7.8 | 33.31 | 6.89 | 72.07 | nasal |
| **YingPterochiroptera** | Hipposideridae | *Doryrhina wollastoni* | 3.88 | 6.93 | 18.39 | 92.03 | nasal |
| **YingPterochiroptera** | Hipposideridae | *Rhinonicteris aurantia* | 15 | 8.99 | 12 | 114 | nasal |
| **YingPterochiroptera** | Hipposideridae | *Paratriaenops furculus* | 9.54 | 5.56 | 8.05 | 98.98 | nasal |
| **YingPterochiroptera** | Hipposideridae | *Triaenops persicus* | 7.48 | 9.1 | 8.43 | 39.82 | nasal |
| **YingPterochiroptera** | Megadermatidae | *Lavia frons* | 24 | 23.8 | 3.5 | 37.5 | nasal |
| **YingPterochiroptera** | Megadermatidae | *Macroderma gigas* | 18.61 | 124.4 | 1.13 | 51.07 | nasal |
| **YingPterochiroptera** | Megadermatidae | *Lyroderma lyra* | 23.29 | 39.27 | 1.39 | 68.25 | nasal |
| **YingPterochiroptera** | Megadermatidae | *Megaderma spasma* | 8.88 | 24.71 | 1.69 | 20.1 | nasal |
| **Yangochiroptera** | Miniopteridae | *Miniopterus australis* | 48.01 | 7.4 | 3.6 | 59.07 | oral |
| **Yangochiroptera** | Miniopteridae | *Miniopterus fraterculus* | 45.8 | 7.38 | 3.16 | 61.92 | oral |
| **Yangochiroptera** | Miniopteridae | *Miniopterus inflatus* | 55 | 14.9 | 2.5 | 47.4 | oral |
| **Yangochiroptera** | Miniopteridae | *Miniopterus manavi* | 44.49 | 7.81 | 2.96 | 54.97 | oral |
| **Yangochiroptera** | Miniopteridae | *Miniopterus natalensis* | 36.29 | 10.43 | 3.48 | 52.07 | oral |
| **Yangochiroptera** | Miniopteridae | *Miniopterus pusillus* | 34.56 | 8.95 | 3.38 | 59.79 | oral |
| **Yangochiroptera** | Miniopteridae | *Miniopterus schreibersii* | 27.11 | 11.46 | 5.47 | 53.41 | oral |
| **Yangochiroptera** | Molossidae | *Chaerephon ansorgei* | 12 | 14.53 | 15 | 17.8 | oral |
| **Yangochiroptera** | Molossidae | *Chaerephon bivittatus* | 14 | 15.42 | 6.8 | 21 | oral |
| **Yangochiroptera** | Molossidae | *Chaerephon chapini* | 8 | 7.49 | 10 | 20 | oral |
| **Yangochiroptera** | Molossidae | *Chaerephon jobensis* | 8 | 20.71 | 10 | 19.8 | oral |
| **Yangochiroptera** | Molossidae | *Chaerephon johorensis* | 9.94 | 15.69 | 5.76 | 19.59 | oral |
| **Yangochiroptera** | Molossidae | *Chaerephon nigeriae* | 10 | 20.14 | 10 | 17 | oral |
| **Yangochiroptera** | Molossidae | *Chaerephon pumilus* | 20.18 | 10.98 | 4.19 | 27.13 | oral |
| **Yangochiroptera** | Molossidae | *Cheiromeles torquatus* | 9 | 169.4 | 14.14 | 27.9 | oral |
| **Yangochiroptera** | Molossidae | *Eumops glaucinus* | 5 | 36.2 | 14.2 | 18 | oral |
| **Yangochiroptera** | Molossidae | *Molossops temminckii* | 10.3 | 5.86 | 7.8 | 50.4 | oral |
| **Yangochiroptera** | Molossidae | *Molossus molossus* | 4.62 | 13.7 | 9.29 | 37.66 | oral |
| **Yangochiroptera** | Molossidae | *Mops condylurus* | 22.09 | 26.59 | 5.57 | 27.12 | oral |
| **Yangochiroptera** | Molossidae | *Mops midas* | 4.8 | 45.5 | 16.5 | 15 | oral |
| **Yangochiroptera** | Molossidae | *Mops mops* | 0.73 | 31.12 | 8.57 | 17.8 | oral |
| **Yangochiroptera** | Molossidae | *Mops niveiventer* | 20.78 | 21.82 | 8.82 | 22.11 | oral |
| **Yangochiroptera** | Molossidae | *Mormopterus jugularis* | 28.42 | 11.56 | 3.98 | 32.04 | oral |
| **Yangochiroptera** | Molossidae | *Ozimops loriae* | 12 | 7 | 6 | 36 | oral |
| **Yangochiroptera** | Molossidae | *Micronomus norfolkensis* | 1.5 | 8 | 8 | 31 | oral |
| **Yangochiroptera** | Molossidae | *Nyctinomops macrotis* | 5 | 16.38 | 15.7 | 17.5 | oral |
| **Yangochiroptera** | Molossidae | *Otomops martiensseni* | 6.4 | 34.92 | 24 | 10.8 | oral |
| **Yangochiroptera** | Molossidae | *Sauromys petrophilus* | 25.87 | 14.25 | 3.74 | 27.99 | oral |
| **Yangochiroptera** | Molossidae | *Tadarida aegyptiaca* | 17.44 | 17.63 | 4.93 | 23.57 | oral |
| **Yangochiroptera** | Molossidae | *Austronomus australis* | 7.36 | 36.4 | 15.52 | 12.77 | oral |
| **Yangochiroptera** | Molossidae | *Tadarida brasiliensis* | 3.16 | 12.61 | 11.25 | 26.72 | oral |
| **Yangochiroptera** | Molossidae | *Tadarida fulminans* | 13 | 33.89 | 15 | 17 | oral |
| **Yangochiroptera** | Molossidae | *Tadarida ventralis* | 4 | 37.43 | 9 | 19.3 | oral |
| **Yangochiroptera** | Mormoopidae | *Mormoops blainvillei* | 16 | 8.69 | 3.16 | 57.5 | oral |
| **Yangochiroptera** | Mormoopidae | *Pteronotus davyi* | 13.03 | 9.52 | 4.76 | 66.22 | oral |
| **Yangochiroptera** | Mormoopidae | *Pteronotus macleayii* | 12 | 12.39 | 4.03 | 69 | oral |
| **Yangochiroptera** | Mormoopidae | *Pteronotus parnellii* | 7.74 | 19.59 | 25.81 | 60.95 | oral |
| **Yangochiroptera** | Mormoopidae | *Pteronotus personatus* | 17.49 | 7.99 | 4.75 | 65.45 | oral |
| **Yangochiroptera** | Mormoopidae | *Pteronotus quadridens* | 15.5 | 5.64 | 3.98 | 81.5 | oral |
| **Yangochiroptera** | Mystacinidae | *Mystacina tuberculata* | 19.97 | 13.14 | 1.94 | 48.38 | oral |
| **Yangochiroptera** | Myzopodidae | *Myzopoda aurita* | 6.72 | 9.1 | 5.32 | 33.4 | oral |
| **Yangochiroptera** | Natalidae | *Natalus stramineus* | 49.56 | 5.68 | 1.58 | 116.48 | oral |
| **Yangochiroptera** | Natalidae | *Nyctiellus lepidus* | 42.7 | 3.88 | 2.7 | 83.4 | oral |
| **Yangochiroptera** | Noctilionidae | *Noctilio leporinus* | 19.47 | 29.93 | 6.46 | 35.27 | oral |
| **Yangochiroptera** | Nycteridae | *Nycteris grandis* | 24 | 29.8 | 3.5 | 20 | nasal |
| **Yangochiroptera** | Nycteridae | *Nycteris macrotis* | 21.55 | 14.49 | 1.18 | 59.97 | nasal |
| **Yangochiroptera** | Nycteridae | *Nycteris thebaica* | 20.43 | 9.2 | 1.41 | 71.32 | nasal |
| **Yangochiroptera** | Phyllostomidae | *Ametrida centurio* | 38.94 | 10.61 | 2.49 | 74.59 | nasal |
| **Yangochiroptera** | Phyllostomidae | *Anoura geoffroyi* | 40.28 | 15.15 | 1.86 | 81.69 | nasal |
| **Yangochiroptera** | Phyllostomidae | *Ardops nichollsi* | 59.79 | 19.23 | 2.39 | 72.59 | nasal |
| **Yangochiroptera** | Phyllostomidae | *Artibeus cinereus* | 41.02 | 12.7 | 1.11 | 60.46 | nasal |
| **Yangochiroptera** | Phyllostomidae | *Artibeus concolor* | 14.73 | 19.65 | 2.92 | 37.21 | nasal |
| **Yangochiroptera** | Phyllostomidae | *Artibeus gnomus* | 33.01 | 10.06 | 1.34 | 60.66 | nasal |
| **Yangochiroptera** | Phyllostomidae | *Artibeus jamaicensis* | 25.98 | 43.63 | 2.32 | 59.25 | nasal |
| **Yangochiroptera** | Phyllostomidae | *Artibeus lituratus* | 24.89 | 59.3 | 1.66 | 62.06 | nasal |
| **Yangochiroptera** | Phyllostomidae | *Artibeus obscurus* | 32.27 | 35.91 | 1.46 | 68.88 | nasal |
| **Yangochiroptera** | Phyllostomidae | *Artibeus phaeotis* | 47.6 | 11.69 | 1.1 | 89.39 | nasal |
| **Yangochiroptera** | Phyllostomidae | *Artibeus toltecus* | 34.38 | 15.47 | 0.81 | 67.15 | nasal |
| **Yangochiroptera** | Phyllostomidae | *Artibeus watsoni* | 22.18 | 11.2 | 0.89 | 62.83 | nasal |
| **Yangochiroptera** | Phyllostomidae | *Brachyphylla cavernarum* | 14.35 | 45.5 | 5.65 | 23.3 | nasal |
| **Yangochiroptera** | Phyllostomidae | *Brachyphylla nana* | 54 | 37.25 | 2.38 | 59 | nasal |
| **Yangochiroptera** | Phyllostomidae | *Carollia castanea* | 26.14 | 13.1 | 0.81 | 95.4 | nasal |
| **Yangochiroptera** | Phyllostomidae | *Carollia perspicillata* | 29.3 | 19.23 | 2.08 | 63.83 | nasal |
| **Yangochiroptera** | Phyllostomidae | *Chiroderma improvisum* | 24.5 | 35.39 | 3.67 | 59.65 | nasal |
| **Yangochiroptera** | Phyllostomidae | *Chiroderma trinitatum* | 23.58 | 13.91 | 3.68 | 86.92 | nasal |
| **Yangochiroptera** | Phyllostomidae | *Chiroderma villosum* | 23.66 | 23.81 | 0.95 | 83.29 | nasal |
| **Yangochiroptera** | Phyllostomidae | *Choeronycteris mexicana* | 35.44 | 17.26 | 2.36 | 50.96 | nasal |
| **Yangochiroptera** | Phyllostomidae | *Desmodus rotundus* | 26.72 | 33.16 | 1.92 | 61.56 | nasal |
| **Yangochiroptera** | Phyllostomidae | *Diaemus youngii* | 21.6 | 36.71 | 2.22 | 51.84 | nasal |
| **Yangochiroptera** | Phyllostomidae | *Ectophylla alba* | 35.47 | 5.55 | 1.27 | 74.58 | nasal |
| **Yangochiroptera** | Phyllostomidae | *Erophylla sezekorni* | 27 | 15.87 | 2.3 | 45.1 | nasal |
| **Yangochiroptera** | Phyllostomidae | *Glossophaga soricina* | 40.68 | 9.97 | 2.28 | 86.48 | nasal |
| **Yangochiroptera** | Phyllostomidae | *Lampronycteris brachyotis* | 4.49 | 10.39 | 0.11 | 98.89 | nasal |
| **Yangochiroptera** | Phyllostomidae | *Leptonycteris curasoae* | 58.31 | 25.27 | 5.39 | 73.5 | nasal |
| **Yangochiroptera** | Phyllostomidae | *Lionycteris spurrelli* | 43.12 | 8.85 | 2.99 | 111.54 | nasal |
| **Yangochiroptera** | Phyllostomidae | *Hsunycteris thomasi* | 32.36 | 7.09 | 1.56 | 97.35 | nasal |
| **Yangochiroptera** | Phyllostomidae | *Lophostoma silvicola* | 23.92 | 32.29 | 1.15 | 66.75 | nasal |
| **Yangochiroptera** | Phyllostomidae | *Macrophyllum macrophyllum* | 20.5 | 8.02 | 2 | 55 | nasal |
| **Yangochiroptera** | Phyllostomidae | *Macrotus waterhousii* | 28.5 | 16.27 | 1.3 | 69.2 | nasal |
| **Yangochiroptera** | Phyllostomidae | *Micronycteris hirsuta* | 28.8 | 12.89 | 1.4 | 80.8 | nasal |
| **Yangochiroptera** | Phyllostomidae | *Micronycteris megalotis* | 34.8 | 6.4 | 1.5 | 98.1 | nasal |
| **Yangochiroptera** | Phyllostomidae | *Micronycteris minuta* | 41 | 6.9 | 1.8 | 82.26 | nasal |
| **Yangochiroptera** | Phyllostomidae | *Gardnerycteris crenulatum* | 20.22 | 13.91 | 1.06 | 69.66 | nasal |
| **Yangochiroptera** | Phyllostomidae | *Monophyllus plethodon* | 47.43 | 15.33 | 2.62 | 96.62 | nasal |
| **Yangochiroptera** | Phyllostomidae | *Phyllonycteris poeyi* | 11 | 15.59 | 4.69 | 39 | nasal |
| **Yangochiroptera** | Phyllostomidae | *Phyllops falcatus* | 31 | 10.82 | 5.57 | 50.5 | nasal |
| **Yangochiroptera** | Phyllostomidae | *Phyllostomus discolor* | 18.14 | 36.7 | 2.25 | 63.16 | nasal |
| **Yangochiroptera** | Phyllostomidae | *Phyllostomus hastatus* | 13.7 | 91.44 | 1.47 | 48.68 | nasal |
| **Yangochiroptera** | Phyllostomidae | *Platyrrhinus brachycephalus* | 40.2 | 14.26 | 4.5 | 91.38 | nasal |
| **Yangochiroptera** | Phyllostomidae | *Platyrrhinus helleri* | 24.67 | 13.44 | 2.83 | 50.87 | nasal |
| **Yangochiroptera** | Phyllostomidae | *Sturnira lilium* | 38.94 | 20.19 | 1.93 | 88.06 | nasal |
| **Yangochiroptera** | Phyllostomidae | *Sturnira ludovici* | 35.62 | 21 | 1.01 | 71.5 | nasal |
| **Yangochiroptera** | Phyllostomidae | *Sturnira mordax* | 29.93 | 11.79 | 1.65 | 72.07 | nasal |
| **Yangochiroptera** | Phyllostomidae | *Sturnira angeli* | 31.59 | 15.46 | 5.46 | 56.48 | nasal |
| **Yangochiroptera** | Phyllostomidae | *Sturnira tildae* | 19.94 | 24.39 | 0.96 | 55.93 | nasal |
| **Yangochiroptera** | Phyllostomidae | *Tonatia bidens* | 19.79 | 27.7 | 1.38 | 78.18 | nasal |
| **Yangochiroptera** | Phyllostomidae | *Trachops cirrhosus* | 24.11 | 36.9 | 1.02 | 64.87 | nasal |
| **Yangochiroptera** | Phyllostomidae | *Trinycteris nicefori* | 25.09 | 8.25 | 0.39 | 82.96 | nasal |
| **Yangochiroptera** | Phyllostomidae | *Uroderma bilobatum* | 28.57 | 16.28 | 1.2 | 92.35 | nasal |
| **Yangochiroptera** | Phyllostomidae | *Vampyrodes caraccioli* | 20.86 | 35.89 | 1.03 | 71.74 | nasal |
| **YingPterochiroptera** | Rhinolophidae | *Rhinolophus acuminatus* | 8.63 | 12.1 | 48.86 | 89.56 | nasal |
| **YingPterochiroptera** | Rhinolophidae | *Rhinolophus affinis* | 15.78 | 13.7 | 27.88 | 80.55 | nasal |
| **YingPterochiroptera** | Rhinolophidae | *Rhinolophus blasii* | 4 | 10.29 | 44.1 | 60.5 | nasal |
| **YingPterochiroptera** | Rhinolophidae | *Rhinolophus capensis* | 12.5 | 12.87 | 42.67 | 84.36 | nasal |
| **YingPterochiroptera** | Rhinolophidae | *Rhinolophus coelophyllus* | 12.5 | 7.07 | 35.01 | 78.46 | nasal |
| **YingPterochiroptera** | Rhinolophidae | *Rhinolophus cornutus* | 13.35 | 7.27 | 43.58 | 105.97 | nasal |
| **YingPterochiroptera** | Rhinolophidae | *Rhinolophus darlingi* | 13.04 | 8.94 | 36.05 | 86.01 | nasal |
| **YingPterochiroptera** | Rhinolophidae | *Rhinolophus denti* | 19.98 | 6.3 | 22.28 | 110.6 | nasal |
| **YingPterochiroptera** | Rhinolophidae | *Rhinolophus euryale* | 15.58 | 9.25 | 21.82 | 105.67 | nasal |
| **YingPterochiroptera** | Rhinolophidae | *Rhinolophus euryotis* | 7.1 | 14.3 | 50.82 | 51.99 | nasal |
| **YingPterochiroptera** | Rhinolophidae | *Rhinolophus ferrumequinum* | 12.55 | 22.59 | 48.09 | 82.18 | nasal |
| **YingPterochiroptera** | Rhinolophidae | *Rhinolophus fumigatus* | 6.64 | 13.09 | 41.27 | 53.24 | nasal |
| **YingPterochiroptera** | Rhinolophidae | *Rhinolophus hildebrandtii* | 5.79 | 25.99 | 46.62 | 34.27 | nasal |
| **YingPterochiroptera** | Rhinolophidae | *Rhinolophus hipposideros* | 14.83 | 4.57 | 40.83 | 109.32 | nasal |
| **YingPterochiroptera** | Rhinolophidae | *Rhinolophus landeri* | 19.2 | 9.39 | 41.85 | 105.41 | nasal |
| **YingPterochiroptera** | Rhinolophidae | *Rhinolophus lepidus* | 18.37 | 5.46 | 35.34 | 103.2 | nasal |
| **YingPterochiroptera** | Rhinolophidae | *Rhinolophus luctus* | 1.49 | 34.07 | 64.97 | 31.07 | nasal |
| **YingPterochiroptera** | Rhinolophidae | *Rhinolophus macrotis* | 6.44 | 6.18 | 28.47 | 46.95 | nasal |
| **YingPterochiroptera** | Rhinolophidae | *Rhinolophus malayanus* | 13.95 | 6.73 | 32.04 | 85.55 | nasal |
| **YingPterochiroptera** | Rhinolophidae | *Rhinolophus megaphyllus* | 7.56 | 10.17 | 56.06 | 68.8 | nasal |
| **YingPterochiroptera** | Rhinolophidae | *Rhinolophus mehelyi* | 5 | 14.03 | 35.9 | 110 | nasal |
| **YingPterochiroptera** | Rhinolophidae | *Rhinolophus monoceros* | 16.27 | 7.49 | 40.39 | 106.45 | nasal |
| **YingPterochiroptera** | Rhinolophidae | *Rhinolophus pearsonii* | 5.33 | 11.55 | 42.3 | 56.68 | nasal |
| **YingPterochiroptera** | Rhinolophidae | *Rhinolophus philippinensis* | 4.71 | 10.91 | 81.84 | 39.78 | nasal |
| **YingPterochiroptera** | Rhinolophidae | *Rhinolophus pusillus* | 12.18 | 5.15 | 36.08 | 109.85 | nasal |
| **YingPterochiroptera** | Rhinolophidae | *Rhinolophus rex* | 3.99 | 32.62 | 43.52 | 26.01 | nasal |
| **YingPterochiroptera** | Rhinolophidae | *Rhinolophus sedulus* | 8.07 | 8.74 | 30.5 | 67.87 | nasal |
| **YingPterochiroptera** | Rhinolophidae | *Rhinolophus shameli* | 7.66 | 9.61 | 34.87 | 69.59 | nasal |
| **YingPterochiroptera** | Rhinolophidae | *Rhinolophus simulator* | 14.59 | 8.13 | 29.37 | 80.71 | nasal |
| **YingPterochiroptera** | Rhinolophidae | *Rhinolophus stheno* | 14.52 | 7.92 | 26.53 | 93.89 | nasal |
| **YingPterochiroptera** | Rhinolophidae | *Rhinolophus swinnyi* | 18.78 | 7.07 | 22.3 | 104.36 | nasal |
| **YingPterochiroptera** | Rhinolophidae | *Rhinolophus thomasi* | 14.8 | 8.26 | 33.89 | 89.53 | nasal |
| **YingPterochiroptera** | Rhinolophidae | *Rhinolophus trifoliatus* | 8.21 | 15.16 | 19.51 | 52.41 | nasal |
| **YingPterochiroptera** | Rhinolophidae | *Rhinolophus yunanensis* | 4.74 | 19.33 | 49.34 | 49.7 | nasal |
| **YingPterochiroptera** | Rhinopomatidae | *Rhinopoma hardwickii* | 4.33 | 13.1 | 9.42 | 32.33 | nasal |
| **YingPterochiroptera** | Rhinopomatidae | *Rhinopoma microphyllum* | 1.88 | 28.02 | 9.88 | 30.72 | nasal |
| **Yangochiroptera** | Thyropteridae | *Thyroptera tricolor* | 24.13 | 4.52 | 2.48 | 54.39 | oral |
| **Yangochiroptera** | Vespertilionidae | *Antrozous pallidus* | 34.36 | 22.24 | 2.59 | 44.76 | oral |
| **Yangochiroptera** | Vespertilionidae | *Barbastella barbastellus* | 17.77 | 8.31 | 3.52 | 35.33 | oral |
| **Yangochiroptera** | Vespertilionidae | *Barbastella leucomelas* | 11.33 | 15.05 | 2.52 | 34.49 | oral |
| **Yangochiroptera** | Vespertilionidae | *Chalinolobus dwyeri* | 15.35 | 8.74 | 6.5 | 24.44 | oral |
| **Yangochiroptera** | Vespertilionidae | *Chalinolobus gouldii* | 18.27 | 14.24 | 9.19 | 29.39 | oral |
| **Yangochiroptera** | Vespertilionidae | *Chalinolobus morio* | 23.69 | 8.91 | 5.35 | 52.92 | oral |
| **Yangochiroptera** | Vespertilionidae | *Chalinolobus picatus* | 12 | 5.86 | 6 | 40 | oral |
| **Yangochiroptera** | Vespertilionidae | *Chalinolobus tuberculatus* | 22.9 | 10.46 | 6.54 | 40.47 | oral |
| **Yangochiroptera** | Cistugidae | *Cistugo lesueuri* | 45.8 | 6.38 | 2.9 | 46.5 | oral |
| **Yangochiroptera** | Cistugidae | *Cistugo seabrae* | 45.15 | 5.37 | 1.99 | 45.92 | oral |
| **Yangochiroptera** | Vespertilionidae | *Corynorhinus townsendii* | 20.33 | 10.3 | 3.28 | 34.73 | oral |
| **Yangochiroptera** | Vespertilionidae | *Eptesicus bottae* | 14.99 | 15.66 | 7.76 | 33.56 | oral |
| **Yangochiroptera** | Vespertilionidae | *Eptesicus brasiliensis* | 20.06 | 9.2 | 9.45 | 34.86 | oral |
| **Yangochiroptera** | Vespertilionidae | *Eptesicus furinalis* | 26.97 | 7.7 | 5.35 | 42.84 | oral |
| **Yangochiroptera** | Vespertilionidae | *Eptesicus fuscus* | 31.56 | 17.49 | 7.93 | 32.55 | oral |
| **Yangochiroptera** | Vespertilionidae | *Eptesicus guadeloupensis* | 13.82 | 18.99 | 6.73 | 26.98 | oral |
| **Yangochiroptera** | Vespertilionidae | *Eptesicus hottentotus* | 32.65 | 30.33 | 5.06 | 30.88 | oral |
| **Yangochiroptera** | Vespertilionidae | *Eptesicus nilssonii* | 9.53 | 10.72 | 11.72 | 28.55 | oral |
| **Yangochiroptera** | Vespertilionidae | *Eptesicus serotinus* | 25.6 | 23.09 | 6.39 | 31.17 | oral |
| **Yangochiroptera** | Vespertilionidae | *Falsistrellus mackenziei* | 35 | 23 | 5 | 40 | oral |
| **Yangochiroptera** | Vespertilionidae | *Falsistrellus tasmaniensis* | 35 | 22.54 | 6 | 36 | oral |
| **Yangochiroptera** | Vespertilionidae | *Glauconycteris variegata* | 34.16 | 11.25 | 2.26 | 40.63 | oral |
| **Yangochiroptera** | Vespertilionidae | *Glischropus tylopus* | 28.31 | 4.59 | 3.43 | 48.16 | oral |
| **Yangochiroptera** | Vespertilionidae | *Hypsugo ariel* | 16.14 | 4.07 | 1.96 | 45.47 | oral |
| **Yangochiroptera** | Vespertilionidae | *Hypsugo bodenheimeri* | 18.09 | 2.73 | 3.97 | 46.91 | oral |
| **Yangochiroptera** | Vespertilionidae | *Hypsugo savii* | 16.7 | 6.3 | 7.08 | 36.15 | oral |
| **Yangochiroptera** | Vespertilionidae | *Idionycteris phyllotis* | 15.83 | 12.13 | 2.92 | 20.37 | oral |
| **Yangochiroptera** | Vespertilionidae | *Kerivoula argentata* | 36 | 10.11 | 2.3 | 92 | oral |
| **Yangochiroptera** | Vespertilionidae | *Kerivoula hardwickii* | 95.09 | 4.55 | 1.99 | 148.17 | oral |
| **Yangochiroptera** | Vespertilionidae | *Kerivoula intermedia* | 69.35 | 3.68 | 2.74 | 83.82 | oral |
| **Yangochiroptera** | Vespertilionidae | *Kerivoula lanosa* | 43.33 | 6.66 | 2.13 | 45.57 | oral |
| **Yangochiroptera** | Vespertilionidae | *Kerivoula minuta* | 47.83 | 2.03 | 1.16 | 105.05 | oral |
| **Yangochiroptera** | Vespertilionidae | *Kerivoula papillosa* | 64.8 | 10.21 | 1.62 | 108.1 | oral |
| **Yangochiroptera** | Vespertilionidae | *Kerivoula pellucida* | 59.03 | 4.14 | 1.86 | 147.27 | oral |
| **Yangochiroptera** | Vespertilionidae | *Laephotis botswanae* | 22 | 7.28 | 5 | 33 | oral |
| **Yangochiroptera** | Vespertilionidae | *Laephotis namibensis* | 13.5 | 8.72 | 2.6 | 22 | oral |
| **Yangochiroptera** | Vespertilionidae | *Laephotis wintoni* | 5.85 | 6.1 | 10.95 | 23.08 | oral |
| **Yangochiroptera** | Vespertilionidae | *Lasionycteris noctivagans* | 24.76 | 11.02 | 9.21 | 30.19 | oral |
| **Yangochiroptera** | Vespertilionidae | *Lasiurus borealis* | 13.84 | 12.33 | 8.5 | 40.95 | oral |
| **Yangochiroptera** | Vespertilionidae | *Dasiypterus intermedius* | 30.41 | 22.96 | 3.18 | 36.18 | oral |
| **Yangochiroptera** | Vespertilionidae | *Murina florium* | 44.82 | 4.41 | 1.01 | 60.51 | oral |
| **Yangochiroptera** | Vespertilionidae | *Murina leucogaster* | 70 | 7.54 | 3 | 70 | oral |
| **Yangochiroptera** | Vespertilionidae | *Murina puta* | 70.19 | 6.91 | 1.49 | 65.99 | oral |
| **Yangochiroptera** | Vespertilionidae | *Murina suilla* | 92.5 | 4 | 1.25 | 124.14 | oral |
| **Yangochiroptera** | Vespertilionidae | *Murina ussuriensis* | 71.59 | 4.69 | 1.84 | 67.14 | oral |
| **Yangochiroptera** | Vespertilionidae | *Myotis albescens* | 44.23 | 5.69 | 1.49 | 73.02 | oral |
| **Yangochiroptera** | Vespertilionidae | *Myotis auriculus* | 59.76 | 38.04 | 3.46 | 49.6 | oral |
| **Yangochiroptera** | Vespertilionidae | *Myotis bechsteinii* | 48.09 | 9.47 | 3.51 | 46.11 | oral |
| **Yangochiroptera** | Vespertilionidae | *Myotis blythii* | 53.72 | 23.82 | 3.54 | 44 | oral |
| **Yangochiroptera** | Vespertilionidae | *Myotis bocagii* | 36.58 | 7.93 | 2.2 | 41.26 | oral |
| **Yangochiroptera** | Vespertilionidae | *Myotis brandtii* | 52.67 | 5.3 | 2.89 | 49.27 | oral |
| **Yangochiroptera** | Vespertilionidae | *Myotis californicus* | 48.61 | 4.39 | 3.29 | 51.44 | oral |
| **Yangochiroptera** | Vespertilionidae | *Myotis capaccinii* | 41.38 | 8.15 | 3.55 | 52.27 | oral |
| **Yangochiroptera** | Vespertilionidae | *Myotis chinensis* | 33.68 | 41.99 | 4.56 | 34.47 | oral |
| **Yangochiroptera** | Vespertilionidae | *Myotis dasycneme* | 32.9 | 15.16 | 4.45 | 39.43 | oral |
| **Yangochiroptera** | Vespertilionidae | *Myotis daubentonii* | 42.78 | 7.63 | 3.26 | 46.98 | oral |
| **Yangochiroptera** | Vespertilionidae | *Myotis dominicensis* | 33.08 | 6.11 | 2.9 | 50.78 | oral |
| **Yangochiroptera** | Vespertilionidae | *Myotis elegans* | 45.92 | 4.21 | 2.61 | 66.28 | oral |
| **Yangochiroptera** | Vespertilionidae | *Myotis emarginatus* | 54.29 | 7.56 | 2.15 | 60.75 | oral |
| **Yangochiroptera** | Vespertilionidae | *Myotis evotis* | 40 | 6.91 | 2.72 | 52 | oral |
| **Yangochiroptera** | Vespertilionidae | *Myotis formosus* | 54.36 | 7.07 | 2.35 | 54.26 | oral |
| **Yangochiroptera** | Vespertilionidae | *Myotis goudotii* | 58.34 | 5.56 | 3.01 | 64.37 | oral |
| **Yangochiroptera** | Vespertilionidae | *Myotis hasseltii* | 42.67 | 8.7 | 5.63 | 47.72 | oral |
| **Yangochiroptera** | Vespertilionidae | *Myotis ikonnikovi* | 59.03 | 5.86 | 2.18 | 48.34 | oral |
| **Yangochiroptera** | Vespertilionidae | *Myotis keaysi* | 45.62 | 5.45 | 2.92 | 62.93 | oral |
| **Yangochiroptera** | Vespertilionidae | *Myotis keenii* | 67.77 | 6.51 | 1.14 | 59.67 | oral |
| **Yangochiroptera** | Vespertilionidae | *Myotis leibii* | 52.9 | 5.22 | 3.77 | 55.78 | oral |
| **Yangochiroptera** | Vespertilionidae | *Myotis longipes* | 39.29 | 7.2 | 5.33 | 44.64 | oral |
| **Yangochiroptera** | Vespertilionidae | *Myotis lucifugus* | 41.15 | 7.8 | 2.52 | 47.39 | oral |
| **Yangochiroptera** | Vespertilionidae | *Myotis macrodactylus* | 41.09 | 7.48 | 4.68 | 51.29 | oral |
| **Yangochiroptera** | Vespertilionidae | *Myotis martiniquensis* | 40.14 | 7.49 | 2.14 | 47.03 | oral |
| **Yangochiroptera** | Vespertilionidae | *Myotis muricola* | 48.1 | 4.8 | 5.07 | 48.69 | oral |
| **Yangochiroptera** | Vespertilionidae | *Myotis myotis* | 38.52 | 25.59 | 5.24 | 35.45 | oral |
| **Yangochiroptera** | Vespertilionidae | *Myotis nattereri* | 67.57 | 7.25 | 3.47 | 46.95 | oral |
| **Yangochiroptera** | Vespertilionidae | *Myotis pilosus* | 39.36 | 26.19 | 3.65 | 40.52 | oral |
| **Yangochiroptera** | Vespertilionidae | *Myotis riparius* | 34.33 | 4.57 | 3.19 | 57.68 | oral |
| **Yangochiroptera** | Vespertilionidae | *Myotis siligorensis* | 49.13 | 2.93 | 4.45 | 79.76 | oral |
| **Yangochiroptera** | Vespertilionidae | *Myotis thysanodes* | 66.97 | 8.49 | 4.01 | 36.52 | oral |
| **Yangochiroptera** | Vespertilionidae | *Myotis tricolor* | 56.91 | 13.67 | 2.16 | 49.12 | oral |
| **Yangochiroptera** | Vespertilionidae | *Myotis velifer* | 37.54 | 9.82 | 2.48 | 43.44 | oral |
| **Yangochiroptera** | Vespertilionidae | *Myotis volans* | 44.71 | 8.71 | 3.71 | 46.39 | oral |
| **Yangochiroptera** | Vespertilionidae | *Myotis welwitschii* | 28.3 | 15.88 | 2.47 | 32.66 | oral |
| **Yangochiroptera** | Vespertilionidae | *Laephotis capensis* | 33.78 | 5.96 | 3.81 | 40.91 | oral |
| **Yangochiroptera** | Vespertilionidae | *Afronycteris nanus* | 22.58 | 3.88 | 4.63 | 69.41 | oral |
| **Yangochiroptera** | Vespertilionidae | *Neoromicia somalica* | 43.3 | 3.53 | 2.15 | 44.98 | oral |
| **Yangochiroptera** | Vespertilionidae | *Neoromicia zuluensis* | 41.14 | 4.14 | 3.06 | 44.89 | oral |
| **Yangochiroptera** | Vespertilionidae | *Nyctalus aviator* | 27.72 | 31.87 | 1.89 | 40.29 | oral |
| **Yangochiroptera** | Vespertilionidae | *Nyctalus lasiopterus* | 5.03 | 45.98 | 22.03 | 15.23 | oral |
| **Yangochiroptera** | Vespertilionidae | *Nyctalus leisleri* | 21.3 | 12.47 | 7.97 | 30.67 | oral |
| **Yangochiroptera** | Vespertilionidae | *Nyctalus noctula* | 10.01 | 28.48 | 13.13 | 22.86 | oral |
| **Yangochiroptera** | Vespertilionidae | *Nycticeinops schlieffenii* | 25.59 | 5.05 | 2.82 | 43.73 | oral |
| **Yangochiroptera** | Vespertilionidae | *Nyctophilus arnhemensis* | 35 | 6.83 | 3 | 51 | oral |
| **Yangochiroptera** | Vespertilionidae | *Nyctophilus geoffroyi* | 45.96 | 8.2 | 4.44 | 49.77 | oral |
| **Yangochiroptera** | Vespertilionidae | *Nyctophilus walkeri* | 26 | 4.44 | 5 | 56 | oral |
| **Yangochiroptera** | Vespertilionidae | *Otonycteris hemprichii* | 28.94 | 21.98 | 4.28 | 37.46 | oral |
| **Yangochiroptera** | Vespertilionidae | *Phoniscus papuensis* | 95 | 6.32 | 3 | 80 | oral |
| **Yangochiroptera** | Vespertilionidae | *Pipistrellus abramus* | 8.83 | 5.87 | 8.33 | 44.6 | oral |
| **Yangochiroptera** | Vespertilionidae | *Parastrellus hesperus* | 33.51 | 3.56 | 3.83 | 43.04 | oral |
| **Yangochiroptera** | Vespertilionidae | *Pipistrellus kuhlii* | 22.15 | 6.07 | 5.18 | 41.16 | oral |
| **Yangochiroptera** | Vespertilionidae | *Pipistrellus maderensis* | 15.35 | 4.91 | 4.53 | 46.44 | oral |
| **Yangochiroptera** | Vespertilionidae | *Pipistrellus nathusii* | 12.71 | 7.44 | 6.66 | 41.4 | oral |
| **Yangochiroptera** | Vespertilionidae | *Pipistrellus pipistrellus* | 23.69 | 5.3 | 5.22 | 47.61 | oral |
| **Yangochiroptera** | Vespertilionidae | *Vansonia rueppellii* | 43.09 | 7.07 | 6.71 | 56.01 | oral |
| **Yangochiroptera** | Vespertilionidae | *Pipistrellus rusticus* | 31.34 | 4.57 | 4.86 | 55.19 | oral |
| **Yangochiroptera** | Vespertilionidae | *Pipistrellus stenopterus* | 25.86 | 15.67 | 7.91 | 38.83 | oral |
| **Yangochiroptera** | Vespertilionidae | *Perimyotis subflavus* | 28.51 | 5.74 | 6.81 | 45.95 | oral |
| **Yangochiroptera** | Vespertilionidae | *Pipistrellus tenuis* | 23 | 3.48 | 7 | 48 | oral |
| **Yangochiroptera** | Vespertilionidae | *Pipistrellus westralis* | 16 | 3.9 | 6 | 46.6 | oral |
| **Yangochiroptera** | Vespertilionidae | *Plecotus auritus* | 25.51 | 8.19 | 2.51 | 36.39 | oral |
| **Yangochiroptera** | Vespertilionidae | *Plecotus austriacus* | 18.98 | 6.75 | 2.94 | 29.66 | oral |
| **Yangochiroptera** | Vespertilionidae | *Rhogeessa tumida* | 34.9 | 4.58 | 5.12 | 48.65 | oral |
| **Yangochiroptera** | Vespertilionidae | *Scoteanax rueppellii* | 40 | 26.41 | 9 | 35 | oral |
| **Yangochiroptera** | Vespertilionidae | *Scotoecus albofuscus* | 26.16 | 4.5 | 2.68 | 39.08 | oral |
| **Yangochiroptera** | Vespertilionidae | *Scotophilus dinganii* | 28.7 | 25.12 | 5.9 | 36.38 | oral |
| **Yangochiroptera** | Vespertilionidae | *Scotophilus leucogaster* | 47.83 | 20.24 | 1.73 | 46.27 | oral |
| **Yangochiroptera** | Vespertilionidae | *Scotophilus nigrita* | 31.21 | 27.34 | 3.92 | 32.85 | oral |
| **Yangochiroptera** | Vespertilionidae | *Scotophilus robustus* | 19.38 | 60.1 | 4.24 | 36.54 | oral |
| **Yangochiroptera** | Vespertilionidae | *Scotophilus viridis* | 28.94 | 19.81 | 3.67 | 40.1 | oral |
| **Yangochiroptera** | Vespertilionidae | *Scotorepens balstoni* | 31 | 11.92 | 8 | 34 | oral |
| **Yangochiroptera** | Vespertilionidae | *Scotorepens greyii* | 10.12 | 10 | 8.57 | 36.08 | oral |
| **Yangochiroptera** | Vespertilionidae | *Scotorepens orion* | 26 | 11.83 | 10 | 36 | oral |
| **Yangochiroptera** | Vespertilionidae | *Scotorepens sanborni* | 17 | 8.13 | 6.3 | 39.3 | oral |
| **Yangochiroptera** | Vespertilionidae | *Tylonycteris pachypus* | 43.73 | 4.1 | 3.76 | 48.95 | oral |
| **Yangochiroptera** | Vespertilionidae | *Tylonycteris robustula* | 61 | 7.98 | 3.78 | 55 | oral |
| **Yangochiroptera** | Vespertilionidae | *Vespadelus baverstocki* | 18 | 4.3 | 4 | 47 | oral |
| **Yangochiroptera** | Vespertilionidae | *Vespadelus caurinus* | 23 | 3.1 | 5 | 59.6 | oral |
| **Yangochiroptera** | Vespertilionidae | *Vespadelus darlingtoni* | 18.6 | 6.06 | 5.02 | 45.71 | oral |
| **Yangochiroptera** | Vespertilionidae | *Vespadelus douglasorum* | 21 | 4.99 | 6 | 52.8 | oral |
| **Yangochiroptera** | Vespertilionidae | *Vespadelus finlaysoni* | 34 | 4.3 | 5 | 35 | oral |
| **Yangochiroptera** | Vespertilionidae | *Vespadelus regulus* | 19.45 | 5.05 | 6.4 | 48.62 | oral |
| **Yangochiroptera** | Vespertilionidae | *Vespadelus troughtoni* | 18 | 5.4 | 6 | 49 | oral |
| **Yangochiroptera** | Vespertilionidae | *Vespadelus vulturnus* | 31.28 | 3.77 | 4.61 | 51.38 | oral |
| **Yangochiroptera** | Vespertilionidae | *Vespertilio murinus* | 8.53 | 15.42 | 14.07 | 24.35 | oral |
| **Yangochiroptera** | Vespertilionidae | *Vespertilio sinensis* | 27.17 | 24.3 | 1.95 | 25.71 | oral |
